# Effects of Zr-Cu Alloy Powder on Microstructure and Properties of Cu Matrix Composite with Highly-Aligned Flake Graphite

**DOI:** 10.3390/ma13245709

**Published:** 2020-12-14

**Authors:** Cunguang Chen, Qianyue Cui, Chengwei Yu, Pei Li, Weihao Han, Junjie Hao

**Affiliations:** 1 Institute for Advanced Materials and Technology, University of Science and Technology Beijing, Beijing 100083, China; cuiqianyue_ustb@163.com (Q.C.); b20160553@xs.ustb.edu.cn (P.L.); b20160508@xs.ustb.edu.cn (W.H.); 2 Innovation Group of Marine Engineering Materials and Corrosion Control, Southern Marine Science and Engineering Guangdong Laboratory (Zhuhai), Zhuhai 519000, China; 3 Beijing Tianyishangjia New Material Corp., Ltd., Beijing 102206, China; yuchengweihaha@163.com

**Keywords:** Cu matrix composite, fake graphite, Zr, thermal conductivity, negative thermal expansion

## Abstract

Highly-aligned flake graphite (FG) reinforced Cu matrix composites with high thermal conductivity and adaptive coefficient of thermal expansion were successfully prepared via the collaborative process of tape-casting and hot-pressing sintering. To overcome the problem of fragile interface, Zr-Cu alloy powder was introduced instead of pure Zr powder to enhance the interfacial strength, ascribed to the physical-chemical bonding at the Cu-FG interface. The results indicate that the synthetic ZrC as interfacial phase affects the properties of FG/Cu composites. The thermal conductivity reaches the maximum value of 608.7 W/m∙K (52% higher than pure Cu) with 0.5 wt % Zr. Surprisingly, the negative coefficient of thermal expansion (CTE) in the Z direction is acquired from −7.61 × 10^−6^ to −1.1 × 10^−6^/K with 0 to 2 wt % Zr due to the physical mechanism of strain-engineering of the thermal expansion. Moreover, the CTE in X-Y plane with Zr addition is 8~10 × 10^−6^/K, meeting the requirements of semiconductor materials. Furthermore, the bending strength of the FG/Cu-2 wt % Zr composite is 42% higher than the FG/Cu composite. Combining excellent thermal conductivity with ultralow thermal expansion make the FG/Cu-Zr composites be a highly promising candidate in the electronic packaging field.

## 1. Introduction

The development of microelectronic products towards miniaturization, multi-function and high integration requires that electronic components have greater power density, which inevitably generates high heat. Thus, the electronic packaging materials must have excellent heat dissipation. In other words, the electronic packaging materials should have high thermal conductivity (TC), coefficient of thermal expansion (CTE) matching with electronic components and good mechanical properties to meet the requirements of heat dissipation and packaging [1,2,3]. Graphite reinforced copper composites have attracted wide attention as potential thermal management materials due to their high thermal performance [4,5,6,7,8,9,10,11]. Graphite materials used as thermally conductive fillers commonly include graphite fiber, flake graphite (FG), and graphite film [4,5,6,12,13,14,15,16,17]. Flake graphite/copper (FG/Cu) composites have good TC (397 W∙m^−1^∙K^−1^) and mechanical properties of Cu, as well as ultra-high TC (1500 W∙m^−1^∙K^−1^), low density, low CTE (−1.0 × 10^−6^ K^−1^ [18]), and good workability of FG [19,20]. Graphite can be used for devices or materials that require directional heat conduction in special cases because of the anisotropy. Generally speaking, FG/Cu composites are prepared by powder metallurgy method and liquid infiltration. However, due to the poor wettability and non-reaction between copper and graphite, the interface of the FG/Cu composites depended on the weak van der Waals force. Therefore, the defects at the interface of the composites would adversely affect the thermal and mechanical properties of the composites.

To solve the above problems, two prevalent approaches of matrix alloying and coatings on carbon surface have been carried out [9,10,15,16,17,21,22,23,24,25,26,27,28]. Matrix alloying was often used in liquid infiltration. Some of the added elements could improve the wettability between carbon and copper by changing the surface tension of liquid copper. Some elements could react with carbon material at the interface and formed a complete carbide layer, which could enhance the interface bonding. Li et al. [24] studied the Zr-containing diamond/Cu composites prepared by the liquid infiltration method, and the TC up to 930 W∙m^−1^∙K^−1^. Remarkably, the TC of the diamond/Cu composite without additives was much lower than that of pure copper, because liquid copper could not wet the diamond surface. The addition of Zr promoted the formation of ZrC at the interface, which facilitated the phonon transfer from the interfacial layer to the diamond. In addition, controlling the thickness of the ZrC layer was extremely important for improving the TC of the diamond/Cu composite. Based on the similar experiments, Wang et al. [21] conducted a more in-depth discussion on the evolution of interface structure. The nucleation of ZrC particles was an inhomogeneous process on the diamond surface, and the morphology of ZrC was strongly influenced by the Zr content. With the appropriate content of Zr (0.5 wt %), the ZrC layers could not only enhance the interface bonding, but also reduce phonon scattering and improve phonon transfer. When the Zr content was higher than 0.5 wt %, the continuous ZrC layer separated the Cu matrix from the diamond. The coatings of metal and carbide on the surface of carbon materials through chemical and physical methods were similar to the layers formed in the matrix alloying, and were commonly used in solid phase sintering and liquid infiltration.

Bai et al. [10] investigated the properties of the flake graphite/Cu composite with the pre-synthesized boron carbide-boron nano-layer on graphite surface. The composition of the nano-layer was mainly boron, with a small amount of B_4_C. The strong chemical bonding effectively improved the bending strength of the composite. However, due to the increase of the interface thermal resistance, the TC and CTE of the composite decreased slightly. It was believed that the homogeneous dispersion and well-controlled alignment of graphite played the most important role in raising the TC of the flake graphite/Cu composite. Besides, the surface coating of materials often has a complicated process, high cost and easy oxidation. However, it is undeniable that the coating could establish a strong chemical bonding at the Cu-graphite interface, effectively bridging the copper matrix and the graphite filler. In conclusion, complete continuous thin layers were formed at the Cu-graphite interface, and the filler was firmly connected to the matrix through chemical bonding. Nevertheless, the thickness and quality of the thin layers were often uncontrollable, leading to the uncertainties in the performance of the composite.

On this occasion, it is not only necessary to improve the interface bonding between the Cu matrix and the FG [27], but also need to excavate the potential of inherent high TC of the FG. In this work, the spot chemical bonding at the Cu-FG interface was considered by introducing the Zr element in the form of Zr-Cu alloy powder, which circumvented the problems that pure Zr powder was easily oxidized to form oxide layers, and was difficult to homogeneously dispersed in the Cu matrix. Simultaneously, a novel technique of tape-casting was adopted to directionally arrange the FGs. Hot-pressing sintering was used to make the FG/Cu composite form a denser structure. The effects of Zr on the microstructure, TC, CTE, and mechanical properties of FG/Cu composites were investigated in detail.

## 2. Materials and Methods 

**Figure 1:**
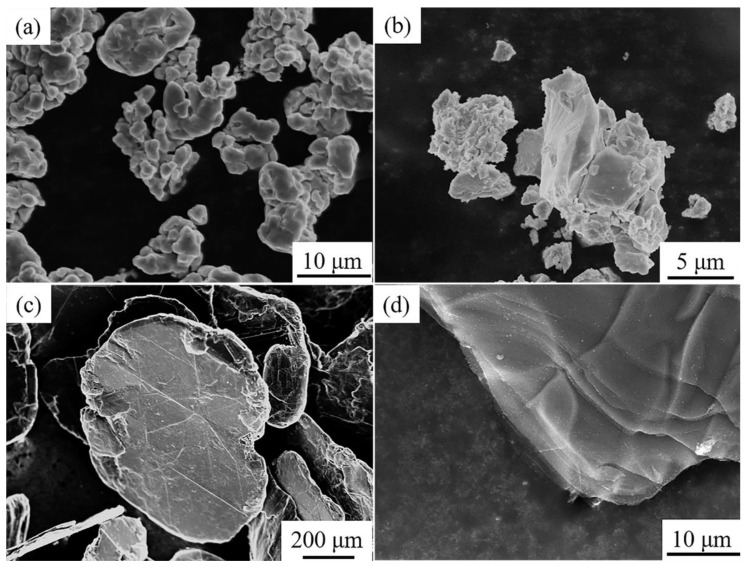
SEM images of raw materials: (**a**) electrolytic copper powder; (**b**) Zr-Cu alloy powders; (**c**,**d**) flake graphite.

**Figure 2:**
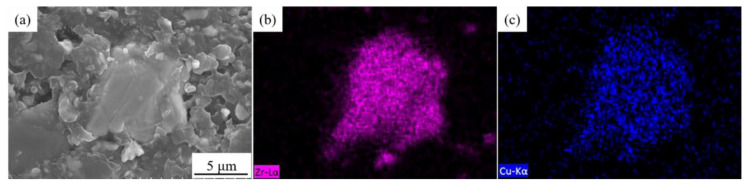
(**a**) SEM image and (**b**,**c**) energy dispersion spectra (EDS) mapping results of Zr and Cu of cross-section of Zr-Cu powder.

Electrolytic pure copper powder (99.9%, D_50_ = 10 μm), Zr-Cu alloy powder with the nominal composition of 74 wt % Zr and 26 wt % Cu, flake graphite (D_50_ = 270 μm, thickness = 50 μm) were the raw materials. The morphology of raw materials is shown in Figure 1. Figure 2 shows the SEM image and energy dispersion spectra (EDS) mapping results of the cross section of Zr-Cu alloy powder. It can be seen that Zr and Cu elements were evenly dispersed without obvious segregation. The EDS results show that the mass fraction of Zr and Cu was 73.59 wt % and 26.41 wt %, respectively. The components of the organic system used for tape-casting included ethanol (analytical reagent), polyvinyl butyral (PVB, analytical reagent), and dibutyl phthalate (DBP, analytical reagent) [28]. The FG/Cu-Zr composites were prepared by the typical method of powder metallurgy.

**Figure 3:**
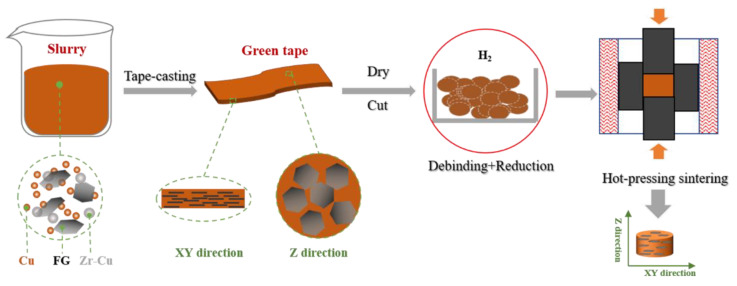
Schematic diagram of the preparation process of flake graphite (FG)/Cu-Zr composite.

The first step was the mixture of the raw powders. Cu powders were mixed with Zr-Cu alloy powders in different proportions (0.5 wt %, 1 wt %, and 2 wt % Zr in the mixing powders) by ball milling for 12 h. The diameter of milling balls was 15 mm, and the weight ratio of ball to powder was 1:3. Then, the pre-mixed powders and 50 vol% FGs were mixed by blender mixer without balls for 3 h. The second step was divided into slurry preparation and tape-casting. The slurry consisted of the composite powder mixture and the organic binders (85 wt % ethanol, 8 wt % PVB, and 7 wt % DBP). The well-stirred slurry was spread out on a plastic substrate. The FG/Cu green tape films were dried at room temperature for 12 h, reduced at 450 °C for 2 h in the H_2_ atmosphere, and cut into wafers. The last step was hot-pressing sintering. The FG/Cu-Zr composites with the dimension of 15 mm in diameter and 10 mm in thickness were prepared by consolidating the wafers in the Ar atmosphere at 1000 °C for 2 h, under the axial pressure of 10 MPa. Figure 3 displays the preparation process of FG/Cu-Zr composites. 

Field emission scanning electron microscope (FE-SEM, HITACHISU 8010, Tokyo, Japan) was used to observe the morphology and interfacial structure of the FG/Cu-Zr composites. Energy dispersion spectra (EDS) attached to the FE-SEM was applied to determine and analyze the elemental composition and distribution. The interfacial configuration and constitution of the composites were characterized by transmission electron microscope (TEM, FEI Tecnai F30, Hillsboro, OR, USA). Phase analysis of the composites relied on X-ray diffraction (XRD, RIGAKUTTR3, Tokyo, Japan). The sample density (ρ) was measured according to the Archimedes’ principle. The thermal diffusivity coefficient of the composite (α) was determined by the laser flash method (NETZSCH LFA447, Selb, Germany). The theoretical specific heat capacity (C_p_) of the composite was calculated based on the mass ratio of three phases. The TC (λ) could be calculated according to the formula: λ = α × C_p_ × ρ. The CTE of the composite was measured by the thermal dilatometer (NETZSCH DIL 402 PC, Selb, Germany) in the range of 25–300 °C. The bending strength of the samples was measured with the method of three-point bending test by the electronic universal testing machine (YHS-216W-200N, Shanghai, China). Here, the in-plane and through-plane locations of the FGs in the composites were defined as the XY and Z directions, respectively.

## 3. Results and Discussion

### 3.1. Microstructure and Phase Composition

**Figure 4:**
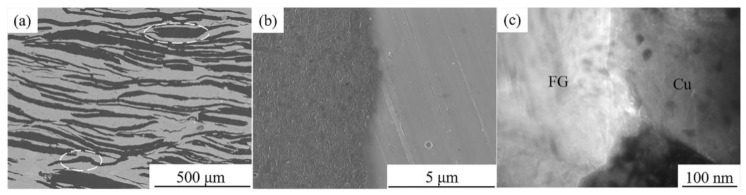
SEM images of (**a**) typical microstructure and (**b**) interface morphology, and (**c**) TEM micrograph of Cu-FG interface of 50 vol% FG/Cu composite.

Figure 4 shows the typical microstructure and the interface structure of the FG/Cu composites. It can be observed that the FGs present highly oriented arrangement in the Cu matrix in Figure 4a. Most of the FGs are stretched in the matrix and are separated from each other by the Cu matrix. A small amount of FGs are stacked and bent as seen in the white dashed area. The soft FGs are prone to deform under the axial pressure during the hot-pressing sintering. However, the softened solid copper provides embedded protection for FGs because of the sintering temperature below the melting point of copper. Therefore, the characteristic feature of this composite is its ability to be easily formed under the sintering pressure of 10 MPa, and the Cu-FG interface bonding is tight without obvious pores as seen in FE-SEM image and TEM micrograph, presented in Figure 4b,c, respectively.

**Figure 5:**
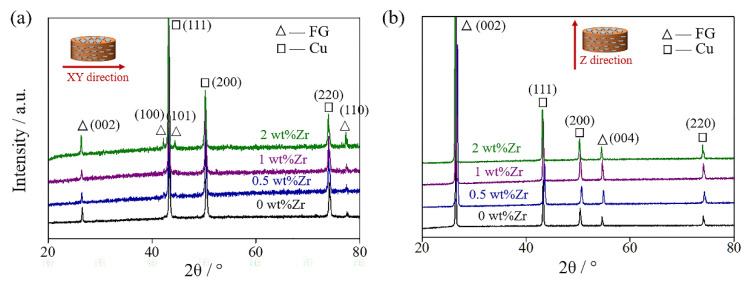
XRD results of FG/Cu composites with different Zr contents in (**a**) XY and (**b**) Z direction, respectively.

Figure 5 displays the XRD patterns of FG/Cu composites with different Zr contents in XY and Z directions (XY-XRD and Z-XRD for short). It can be found that the XY-XRD diffraction peak of graphite is extremely strong, while the Z-XRD diffraction peak of (004) crystal plane is too weak to be observed. Presumably, the FGs with special lamellar structure are highly oriented in the matrix, thus obtaining the highly recognizable XRD results. Besides, no significant difference in the XRD patterns with various Zr contents is detected, which could be attributed to the low content of Zr and its compounds in the composites.

**Figure 6:**
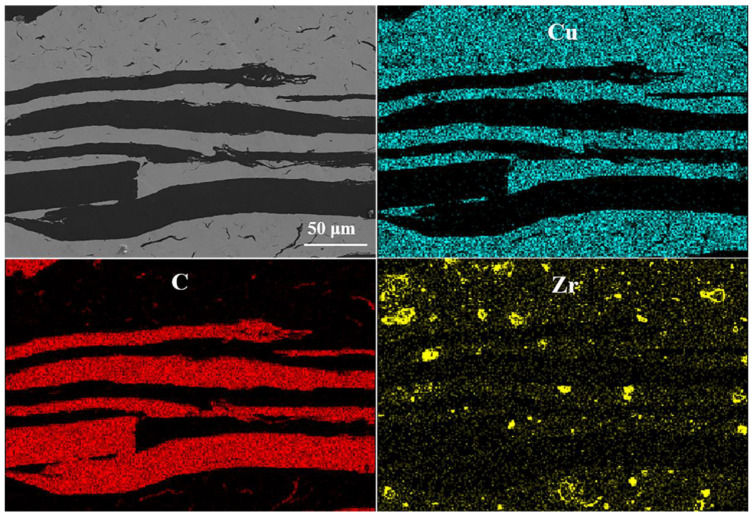
Element distribution mapping of FG/Cu composite with 2 wt % Zr.

**Figure 7:**
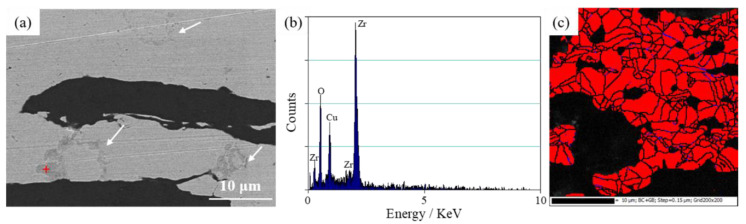
(**a**) SEM image indicating Zr circles in the Cu matrix by white arrows; (**b**) EDS spectrum corresponding to red cross in (**a**); (**c**) EBSD result of FG/Cu composite with 2 wt % Zr. Blue spots represent residual carbon in (**c**).

Element distribution mapping of the longitudinal section of the FG/Cu composite with 2 wt % Zr (FG/Cu-2Zr) shows the approximate distribution of Zr element in the composite as seen in Figure 6. The FG/Cu-2Zr composite is selected as the representative for Zr with the high content in the composite is easier to be detected by EDS. It can be observed that Zr is dispersed in the Cu matrix and the Cu-FG interface like coarse particles. Due to the thorough ball-milling, the Zr-Cu particles were randomly dispersed in the matrix. Inevitably, high temperature made for the chemical reaction between FG and Zr existed at the Cu-FG interface. Dramatically, the Zr in the matrix looked like circles as shown in Figure 7. According to the Cu-Zr phase diagram [29], Zr has an extremely low solid solubility in Cu even at high temperature. It is almost impossible for Zr to form a solid solution in Cu matrix through atomic diffusion during the solid-phase sintering, such as hot-pressing sintering. Compared with the continuous Zr layers in the diamond/Cu-Zr composite prepared by gas pressure infiltration [21], it is reasonable that no continuous Zr layers exist in the FG/Cu-Zr composites prepared by the modified method of powder metallurgy. It can be speculated that the interface phase is composed of Zr, Cu, FG and new phase formed by the chemical reaction. Previous studies reported that the reaction diffusion between Cu and Zr was easily occurred at high temperature, forming intermetallic compounds (such as Cu_9_Zr_2_, Cu_51_Zr_14_, Cu_8_Zr_3_, etc.) [30]. Generally, compared with a complete crystal, the surface and interface of crystals have higher diffusion coefficient, which attributes to the irregular arrangement of atoms on these defects. The numerous grain boundaries between copper grains are excellent channels for high diffusivity in the matrix of the composite. As a result, the free-dispersed Zr in the copper matrix diffused and reacted with Cu along the grain boundary at high temperature, generating Zr circles in the 2D projection as pointed out by white arrows in Figure 7a. The grain size of Cu matrix is less than 10 μm as seen in Figure 7c, which are much smaller than our previous study (greater than 20 μm) [28]. Combined with the small amount of residual carbon (blue dots) detected by EBSD, it could be suggested that the residual carbon contributes to the refined grains of copper matrix.

**Figure 8:**
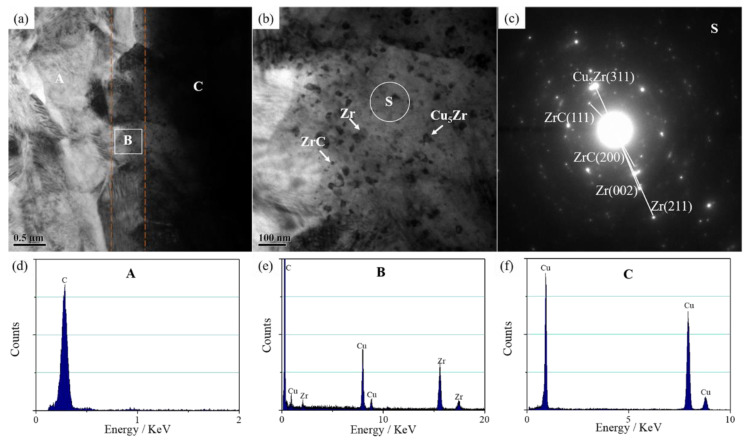
(**a**) Typical TEM image of interface in FG/Cu-2Zr composite; (**b**) magnified detail image of white rectangle in (**a**); (**c**) the electron diffraction pattern and analysis of the marked area S in (**b**); (**d**–**f**) EDS data of the marked points A, B, and C in (**a**), respectively.

To ascertain the existing form of Zr in the composite, TEM was used to obtain the microstructure and diffraction spots of the FG/Cu-2Zr composite as seen in Figure 8. Macroscopically, an aggregate composed of Zr looks like large particles shown in Figure 6 and Figure 7a. Nevertheless, some nano-scale particles scattered at the interface under TEM at high magnification. Analysis of diffraction spot of the submicron particles reveals the complex composition, including Zr, ZrC, and Cu_5_Zr. The reaction diffusion between Cu and Zr-Cu alloy, as well as the chemical reaction between FG and Zr (Equation (1)) during the high-temperature sintering contributed to the mixed nanoparticles.

(1) Zr+C→ZrC

**Figure 9:**
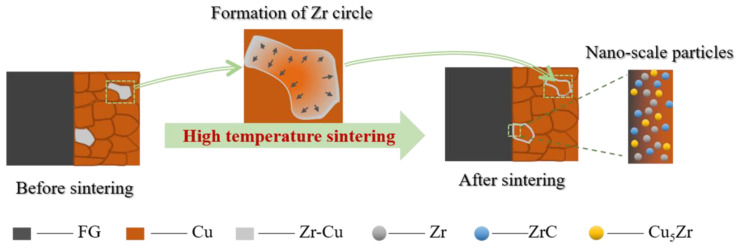
Morphological evolution of Zr in the FG/Cu composite.

The results suggest that the transformation of interface combination is completed from physical bonding to physical–chemical hybrid bonding. The related schematic diagram is shown in Figure 9, which clearly illustrates the existence and distribution of Zr in the Cu matrix and the interface after the high temperature sintering.

### 3.2. Thermal Properties

The thermal properties of the FG/Cu composites with various Zr contents is listed in Table 1. As the Zr content rises from 0 wt % to 2 wt %, the TC in the XY direction (XY-TC for short) of the composites increases first and then decreases. The maximum TC reaches 608.7 W/m∙K with 0.5 wt % Zr. The introduction of 0.5 wt % Zr slightly enhanced the XY-TC of the FG/Cu composite, which could attribute to the change of the bonding way. The ZrC generated at the Cu-FG interface, and changed the bonding mode from pure physical bonding to physical–chemical cooperative bonding, reducing the microcracks that were difficult to be observed at the interface of the composite. Therefore, the phonon scattering and phonon transmission at the Cu-FG interface were weakened and potentiated, respectively. Nevertheless, further increasing of Zr content (higher than 0.5 wt %) would increase the ZrC content at the interface, which was equivalent to increasing the thickness of the interface layer. Besides, the more reaction between Zr and FG at the interface, the more chemical damage of the FG surface. The thickened interface layer plus the damaged graphite surface aggravated the phonon scattering and increased the interface thermal resistance, reducing the TC of the FG/Cu-Zr composite. Consequently, controlling the Zr addition in the composite is essential to enhance the TC of the composite.

The CTE values of the FG/Cu composites with different Zr contents in the XY direction and Z direction were measured in range of 25 °C to 300 °C. The CTE in the XY direction (XY-CTE) of the composite decreased from 14.63 × 10^−6^/K to 12.76 × 10^−6^/K with the addition of Zr. Compared with the predicted values of the rule of mixture (8 × 10^−6^/K), Turner model (2.94 × 10^−6^/K) [31], and Kerner model (6.95 × 10^−6^/K) [32], the measured value of the XY-CTE was much higher. Ren et al. [9] believed that increasing the effective contact area between the FGs and the Cu matrix was essential to exploit the advantage of the low CTE of FGs in the XY direction. However, the tape-casting method aligned the FGs in the same direction, resulting in lower effective contact areas between FGs and the matrix. As well known, due to the difference in CTE between Cu and FGs, the thermal stress generates at the Cu-FG interface after the composite is heated. The interface layer without a strong chemical bonding is unable to resist the shear stress caused by the expansion of the Cu matrix, restraining the advantage of low CTE of FGs. The addition of Zr introduced a spot chemical bonding to the Cu-FG interface, improving the interfacial strength of the composite. The obtained lower XY-CTE of the FG/Cu-Zr composites benefited from the increased effective contact area, which ascribed the strengthened interface. As reported in our previous study [28], it is found that the interlayer sliding fracture of FGs was an important reason for the large XY-CTE.

It can be observed in Table 1 that the Z-CTE of the composite increases with the Zr addition. It is noteworthy that the Z-CTE exhibits a negative thermal expansion. Regarding the anomalous thermal expansion in the Z direction, Firkowska et al. [7] believed that the residual strain in the composite induced the temperature-dependent in-plane strain in the graphite, leading to a shrinking through-plane lattice constant of the graphite. In other words, the Z-CTE of the graphite changed. Based on the elastic theory, Firkowska obtained the effective Z-CTE of the graphite platelets by calculation, which was reduced from 28 × 10^−6^/K to −26 × 10^−6^/K. 

**Figure 10:**
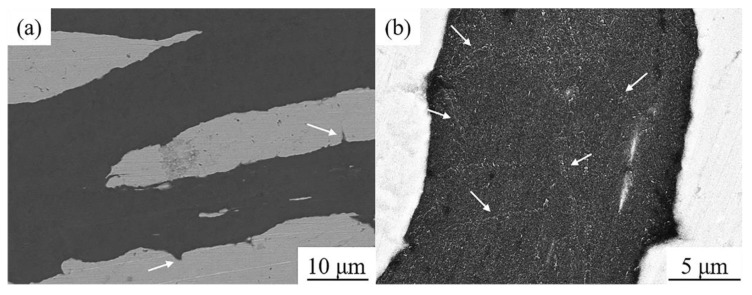
Wrinkles on the (**a**) surface and (**b**) inside of the FGs.

**Figure 11:**
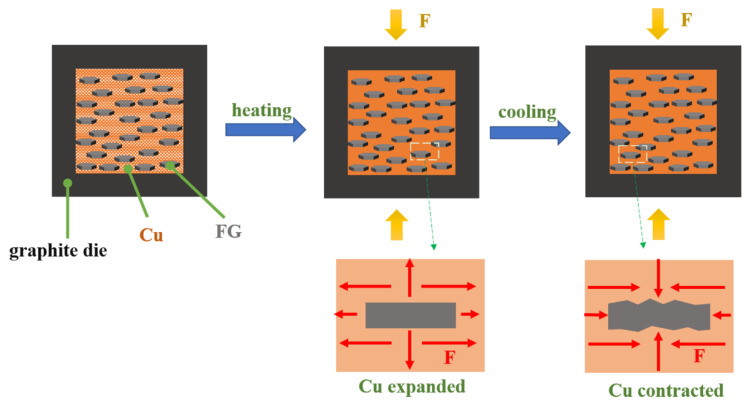
Forming process of the wrinkles on the FG surface.

On the macroscopic perspective, some wrinkles appear on the inside surface of the FGs are discovered in this work as shown in Figure 10. Figure 11 briefly describes the formation process of the wrinkles. The Cu matrix expands with elevated temperature during the sintering process, while the thermal expansion of FGs is much smaller. After sintering, the Cu matrix contracts with temperature falling, bringing about the pressure on the FGs along the XY direction. The FGs deforms under the pressure, increasing the thickness of the FGs intuitively. Furthermore, due to the pressure cooling method, the shrinkage rate of the Cu matrix slows down, reducing the shear force on the FGs. Therefore, the decreased deformation of FGs avoids the structural integrity destruction of the FGs. It is worth mentioning that wrinkles on the FG surface could increase the friction force of the Cu-GF interface, which is beneficial to increase the effective contact area of the interface. Oddone et al. [33] believed that FG surface wrinkles can be straightened when heated again. Accordingly, the thermal expansion of the Cu matrix in the CTE testing of the composite stretches the wrinkles of the FG, reducing the thickness of the FG. Eventually, the thermal expansion behavior of the composite in the Z direction can be considered as the superposed results of the expansion of the Cu matrix and the contraction of the FGs. In addition to affecting the interface, Zr also affects the Z-CTE of the composite by influencing residual thermal stress and the CTE of the Cu matrix. The CTE of Zr is approximately 3.1 × 10^−6^/K, and the granular Zr dispersed in the Cu matrix could slightly reduce the CTE of the matrix. It could be inferred that the reduced CTE of the matrix decreases the residual stress induced by the mismatch of thermal expansion. As a result, the shrinkage of the FG in the Z direction decreases, that is, the effective Z-CTE of the FG increases.

### 3.3. Mechanical Properties

**Figure 12:**
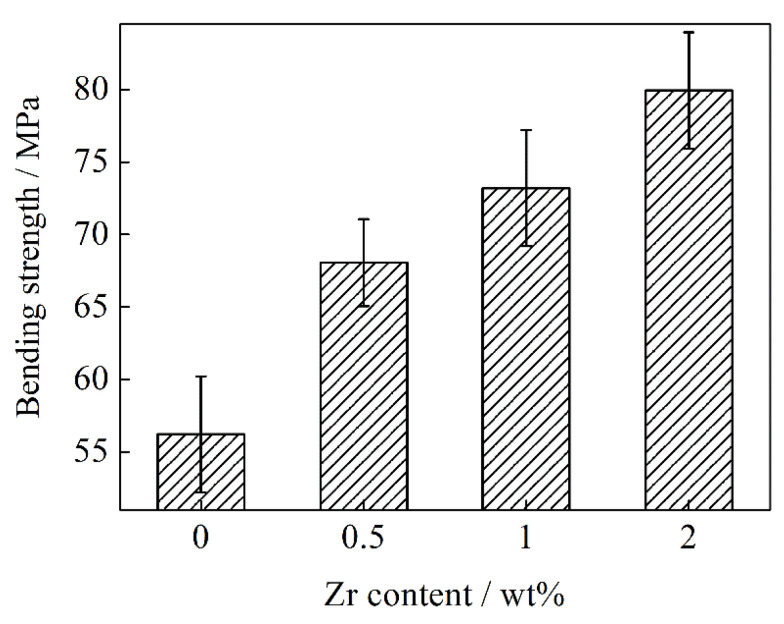
Bending strength of FG/Cu composites with different Zr contents.

Figure 12 represents the bending strength of the FG/Cu composites with different Zr contents. It is noted that the force was applied along the height of the samples, that is, the Z direction of FGs. The bending strength of the FG/Cu-Zr composites increases from 56.2 MPa to 79.9 MPa as the Zr content increases from 0 wt % to 2 wt %, indicating that the addition of Zr is beneficial to improving the mechanical performance of the FG/Cu composite. On the one hand, ZrC is formed at the Cu-FG interface, strengthening the interface bonding and optimizing the interface structure. It is indicated that Zr has an imperfect diffusion in Cu matrix as seen in Figure 6 and Figure 7a. Due to the excessive addition of Zr in the matrix, non-solution Zr exists as pure Zr particles and Cu_5_Zr intermetallic compound particles. It was reported that nano and submicron particles had the dispersion strengthening on Cu [34,35]. Therefore, it can be inferred that the incompletely diffused nano Zr and Cu_5_Zr particles locally strengthen the Cu matrix, which improves the overall mechanical property of the composite.

**Figure 13:**
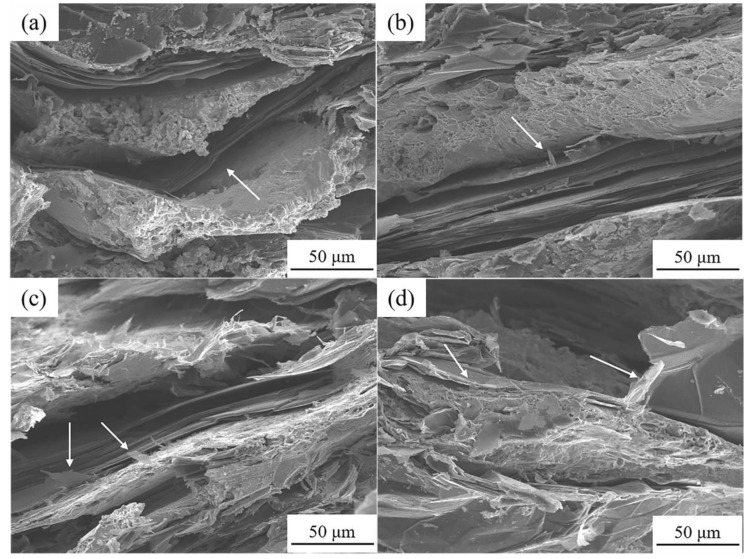
Fracture appearance of FG/Cu-Zr composites with (**a**) 0 wt %, (**b**) 0.5 wt %, (**c**) 1 wt %, and (**d**) 2 wt % Zr.

Figure 13 shows the fracture morphology of the FG/Cu composites with different Zr contents. It can be found that the FGs are detached from the Cu matrix and accompanied by fragmentation phenomena in the FG/Cu-0Zr composite as seen in Figure 13a. However, graphitic layers near the Cu matrix peel off, and the part far from the matrix is still intact in the FG/Cu composites with 0.5 wt % and 1 wt % Zr as seen in Figure 13b,c. With 2 wt % Zr (Figure 13d), the graphite crystal near the Cu-FG interface is destroyed, aggravating the disintegration of the flake graphite near the interface. As mentioned earlier, the added Zr in the composite can react with the graphite at the Cu-FG interface, improving the interface bonding force. Thus, it can be deduced that the fracture does not occur at the interface, but between the graphite layers, which suggests that the addition of Zr effectively transferred the breaking load from Cu-FG interface to the FG layers.

In general, due to the immiscibility and non-reaction of Cu and C, the poor Cu-FG interface is detrimental to the thermal and mechanical properties of the composite. Interestingly, the introduction of Zr in this work enhances both the thermal and mechanical properties of the composite, which should be attributed to the formation of the strong Cu-ZrC-FG interface bonding. 

The performance of FG/Cu composites in various literatures is summarized in Table 2. It can be found that the pressure sintering is commonly chosen to prepare FG/Cu composites, owing to its ability to obtain the tightly integrated Cu-FG interface. Higher TC acquired in this study should give the credit to the high alignment of FGs in the matrix acquired by a special process [7,10,18,36,37]. CTE in this study has wider adjustment range when TC is not prominent [9]. Compared with the research data in various studies, the comprehensive properties of the FG/Cu composite in this study is inspiring so that it can be regarded as a highly promising candidate in the electronic packaging field.

## 4. Conclusions

1. The flake graphite/Cu composites with superior thermal properties were successfully prepared via tape-casting and hot-pressing sintering. The flakes graphite showed high alignment in the Cu matrix, significantly improving the TC of FG/Cu-Zr composites. The added Zr acted with the FGs at the interface and the Cu matrix, forming ZrC and Cu-Zr intermetallic compounds, respectively.
2. The maximum value of the TC of the FG/Cu composite reached 608.7 W/m∙K with 0.5 wt % Zr. The TC of the composite decreased with increasing the Zr content.
3. The XY-CTE of the FG/Cu composite decreased as the Zr content increased, which attributed to the greater effective contact area caused by the interface strengthening. The addition of Zr weakened the negative thermal expansion in the Z direction of the composite, which was occasioned by the decreased thermal stress of the composite.
4. The bending strength of the FG/Cu composite increased with the Zr addition. The bending strength of the FG/Cu composite with 2 wt % Zr reached 79.9 MPa, 42% higher than the composite without Zr. The exfoliation of graphite layers near the FG-Cu interface caused the fracture of the FG/Cu composites. All in all, the FG/Cu composite with 0.5 wt % Zr showed the best overall performance, including the thermal and mechanical properties.

## Figures and Tables

**Table 1:** Thermal properties of FG/Cu composites with various Zr contents.

Zr Content (wt %)	ρ (g/cm^3^)	C_p_ (J/g·K)	α (mm^2^/s)		λ (W/m∙K)		CTE (×10^−6^/K)	
			XY	Z	XY	Z	XY	Z
0	5.47 ± 0.1	0.452	241.2 ± 1.5	20.3 ± 0.4	596.5 ± 3.7	50.2 ± 1.0	12.5	−7.61
0.5	5.43 ± 0.1	0.452	248.0 ± 1.4	17.5 ± 0.3	608.7 ± 3.4	43.0 ± 0.7	9.63	−4.34
1	5.39 ± 0.1	0.452	229.0 ± 1.0	15.7 ± 0.5	558.0 ± 2.4	38.2 ± 1.2	8.56	−3.46
2	5.36 ± 0.1	0.452	216.6 ± 1.4	15.3 ± 0.4	524.8 ± 3.4	37.1 ± 1.0	9.58	−1.1

**Table 2:** Comparisons of the thermal conductivity (λ), coefficient of thermal expansion (CTE), and bending strength of FG/Cu composites in this study and previous reports.

FG Fraction (vol%)	Interfacial Layer	Fabrication Method	λ (W/m∙K)		CTE (×10^−6^/K)		Bending Strength (MPa)	Reference
			XY	Z	XY	Z		
50	ZrC	HP ^a^	608.7	43	9.63	−4.34	68	This study
50	Cr_3_C_2_	HP	628	80	14.5	5	93	[9]
70	B_4_C	HP	608	40	5.2	3.9	72	[10]
50	SiC	HP	491	66	/	/	79	[37]
50	Mo_2_C	HP	598	42	13.9	−2.92	75	[28]
50	/	SPS ^b^	503	/	/	2	/	[7]
40	/	RA ^c^ + HP	640	100	/	/	/	[5]
30	/	VF ^d^ + SPS	438	80	12	6.2	/	[38]
35	/	VF + SPS	525	106	/	/	/	[39]
60	Ni	HP	532	/	15	−3.85	75	[18]
51	/	HP	488	96	7.5	/	/	[36]

^a^ hot-pressing sintering; ^b^ spark plasma sintering; ^c^ resonant acoustic mixer; ^d^ vacuum filtration method.
